# Identification of Novel Parishin Compounds from the Twig of *Maclura tricuspidata* and Comparative Analysis of Parishin Derivatives in Different Parts

**DOI:** 10.3390/molecules28010007

**Published:** 2022-12-20

**Authors:** Dae-Woon Kim, Jong-Kuk Kim, Yoseph Asmelash Gebru, Young-Hoi Kim, Han-Seok Choi, Myung-Kon Kim

**Affiliations:** 1 Department of Food Science and Biotechnology, Jeonbuk National University, Jeonju 54896, Republic of Korea; 2 Institute of Jinan Red Ginseng, Jinan-gun 55442, Republic of Korea; 3 Department of Biological and Chemical Engineering, Mekelle University, Mekelle 231, Ethiopia; 4 Department of Agriculture and Fisheries Processing, Korea National College of Agriculture and Fisheries, Jeonju 54874, Republic of Korea

**Keywords:** *Maclura tricuspidata*, twig, parishin derivatives, macluraparishins, 3-hydroxy-3-methylpentane-1,5-dioic acid, different parts, quantitation

## Abstract

Parishin compounds are rare polyphenolic glucosides mainly found in the rhizome of the traditional Chinese medicinal plant, *Gastrodia elata*. These constituents are reported to have several biological and pharmacological activities. In the present study, two novel parishin derivatives not previously reported as plant-based phytochemicals were identified from a twig of *Maclura tricuspidata* (MT) and two new compounds were elucidated as 1-(4-(β-d-glucopyranosyloxy)benzyl)-3-hydroxy-3-methylpentane-1,5-dioate (named macluraparishin E) and 1,3-bis(4-(β-d-glucopyranosyloxy)benzyl)-3-hydroxy-3-methylpentane- 1,5-dioate (macluraparishin C), based on the experimental data obtained by UV–Visible (UV–Vis) spectroscopy, high performance liquid chromatography–quadrupole time-of-flight mass spectrometry (HPLC-QTOF-MS) and nuclear magnetic resonance (NMR) spectroscopy. Additionally, gastrodin, parishin A and parishin B were positively identified by spectroscopic evidence and the comparison of HPLC retention time with the corresponding authentic standards. Gastrodin, parishin A and parishin B, macluraparishin E and macluraparishin C were found to be the most abundant constituents in the MT twig. The compositions and contents of these constituents were found to vary depending on the different parts of the MT plant. In particular, the contents of parishin A, parishin B, macluraparishin C and macluraparishin E were higher in the twig, bark and root than in the leaves, xylem and fruit.

## 1. Introduction

*Maclura tricuspidata* (carr.) Bur, belonging to the Moraceae family, is a thorny deciduous tree distributed throughout the East Asian region including Korea, China and Japan. In the Korean traditional medical books such as Donguibogam (1613 A.D. Joseon Dynasty), most parts of this plant have been utilized as folk medicine for the treatment of various disorders such as neuritis, arthritis, mumps, tuberculosis, inflammation, jaundice and hepatitis [1,2]. Recent research results have also reported that MT extract has several beneficial health effects, including anticancer [3,4], anti-inflammatory [5], antioxidant [6,7], antiobesity and antidiabetic effects [8]. 

Several bioactive prenylated xanthones, phenolic acids and flavonoids have been previously identified from the stem, stem bark, root, leaves or fruit of MT [2,9,10]. These compounds were also reported to have antibacterial, antifungal [11], antitumor [12,13], antioxidant [14,15,16], neuroprotective [17], cytotoxic [14,18,19], anti-inflammatory [2,20,21], hepatoprotective [22], gastroprotective [20] and α-glucosidase inhibition activities [23]. However, the screening of natural products, particularly those with new chemical structures has been recognized as an important process for the discovery of new biologically and pharmacologically active constituents. In our previous study [24], we reported that MT fruit contains several parishin-related compounds (gastrodin, parishins A, B, C and E) based on tentative identification results using HPLC-QTOF-MS. This finding incited us to conduct further studies to identify the more detailed chemical structures of the parishin-related compounds in the MT plant. Parishin compounds are well-known bioactive principles in the rhizome of *G. elata* Blume. The *G. elata* rhizome has traditionally been used for the prevention and treatment of central nervous system (CNS) diseases such as dizziness, insomnia, stroke, spasm, amnesia, sedative, hypnotic, headaches and convulsions [25,26,27] in traditional Korean medicine. It has been reported that various bioactivities of *G. elata* rhizome are mainly due to the presence of parishin and its derivatives. Parishins A, B, C and E are tri- di- or monoester compounds of citric acid and gastrodin (4-β-d-(glucopyranosyloxy)benzyl alcohol). Parishin and its derivatives can be metabolized into intermediates or final metabolites such as gastrodin and 4-HBA in vitro and in vivo systems [25,28]. Gastrodin and 4-HBA possess a wide range of beneficial health effects for disorders of the central nervous system such as epilepsy, Alzheimer′s and Parkinson′s diseases, cognitive impairment and cerebral ischemia [25,27,28,29]. Nevertheless, little is known about the parishin-related compounds in the MT twig and other different parts of this plant. In this study, we report on the isolation and identification of novel parishin derivatives, which have never been reported previously, as plant-based phytochemicals with four known compounds in the MT twig and their composition and contents in different parts of the MT plant.

## 2. Results and Discussions

### 2.1. HPLC-QTOF-MS Analysis of MT Twig Extract

**Figure 1:**
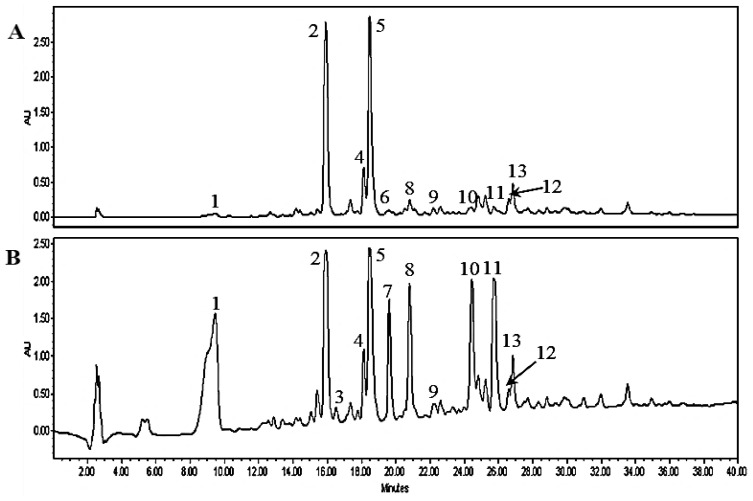
HPLC-PDA profiles of 70% MeOH extract of *M. tricuspidata* twig. (**A**) 280 nm; (**B**) 220 nm. **1**, gastrodin; **2**, taxifolin-7-β-d-glucoside; **3**, parishin E; **4**, chlorogenic acid; **5**, dihydrokaempferol-7-β-d-glucoside; 6, quercetin-3-β-d-glucoside; **7**, unknown-1; **8**, parishin B; **9**, parishin C; **10**, parishin A; **11**, unknown-2; **12**, dihydrokaempferol; **13**, kaempferol-3-β-d-glucoside.

Although HPLC coupled with photodiode array detector (PDA) is the most common method for the detection of polyphenolic compounds, these compound groups have very different maximum absorption wavelengths (λ_max_) according to their characteristic chromophores [30]. Therefore, the proper wavelength should be set to detect targeted polyphenolic compounds in the matrix. Figure 1 shows HPLC chromatograms at 280 and 220 nm of 70% methanol extract of MT twig, respectively. The chromatogram monitored at 220 nm (Figure 1B) showed several large peaks, whereas the chromatogram at 280 nm (Figure 1A) showed only two large peaks with a few small peaks. These results indicate that the desirable wavelength for the analysis of polyphenolic compounds in TM twig extract is 220 nm rather than 280 nm. Plant-based secondary metabolites can be analyzed with various methods. Among them, The QTOF-MS method coupled with HPLC or ultra-performance liquid chromatography (UPLC) is being widely applied to identify trace constituents which are not detectable by the classical methods due to its high resolution, accurate mass measurement and high sensitivity [31]. In this study, the MT twig extract was analyzed by HPLC-QTOF-MS in negative electrospray ionization (ESI) mode, whereby the detected constituents were characterized by the interpretation of their mass spectra and the searching of library databases.

**Figure 2:**
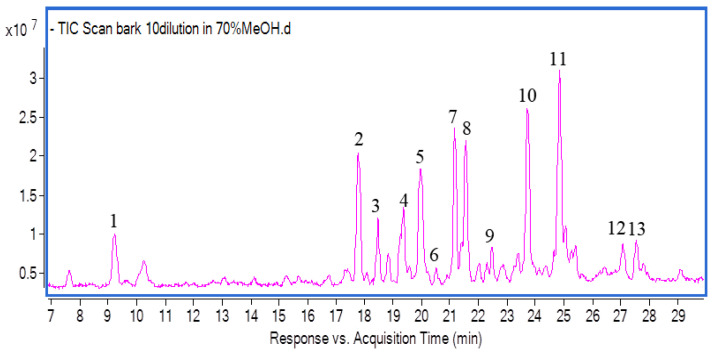
Total ion chromatograms of 70% MeOH extract of MT twig by HPLC-QTOF-MS. Peak numbers refer to Table 1.

The total ion current (TIC) chromatogram obtained by HPLC-QTOF-MS coupled with PDA (at 220 nm) of the MT twig extract is shown in Figure 2. The characteristics of the identified compounds are listed in Table 1 with their molecular formula, molecular mass and error values (ppm) calculated by the software. As phenolic compounds, peak **2** yielded a deprotonated molecular ion peak at *m*/*z* 465.1031 [M − H]^−^ with a fragment ion at *m*/*z* 303.0505 [M – H − glucose + H_2_O]^−^ due to loss of a glucosyl moiety. This compound was tentatively characterized as a taxifolin-7-β-d-glucoside. Peak 4 showed a deprotonated molecular ion peak [M − H]^−^ at *m*/*z* 353.0872 with *m*/*z* 191.0558 [M − H − caffeic acid + H_2_O]^−^ due to the loss of a caffeoyl moiety. This compound was identified as a chlorogenic acid.

Peak **5** showed deprotonated molecular ion peaks at *m*/*z* 449.1082 [M − H]^−^ with *m*/*z* 287.0558 [M − H − glucose + H_2_O]^−^due to loss of a glucosyl moiety. Accordingly, this compound was identified as dihydrokaempferol-7-β-d-glucoside. Peak **6** showed a deprotonated molecular ion peak at *m*/*z* 625.1405 [M − H]^−^ corresponding to quercetin-3-sophoroside. Peaks **12** and **13** were tentatively identified as dihydrokaempferol and kaempferol-3-β-d-glucoside based on deprotonated molecular ion peaks [M − H]^−^ at *m*/*z* 286.0558 and *m*/*z* 447.0927, respectively. These compounds have been previously reported as chemical constituents of the MT plant [2,32,33].

Seven compounds (peaks **1, 3** and **7**–**11**) can be considered as parishin-related compounds (λ_max_ 268 nm) because their UV profiles were very similar to those of parishin A (λ_max_ 268 nm). Peaks **1** and **3** were identified as gastrodin (4-(β-d-glucopyranosyloxy)benzyl alcohol) and parishin E (2-[4-O-(β-d-glucopyranosyl)benzyl] citrate) based on deprotonated molecular ion peaks observed at *m*/*z* 331.1030 [M − H + HCOOH]^−^ and *m*/*z* 459.1138 [M − H]^−^, respectively. Peaks **8** and **9** showed deprotonated molecular ion peaks [M − H]^−^ at *m*/*z* 727.2085 and *m*/*z* 727.2086, respectively. Peaks **8** and **9** were identified as parishin B and parishin C by comparison of the QTOF-MS spectral data with references [24,34,35] and retention time with authentic standards. Peak **10** was identified as parishin A by the interpretation of a fragment ion at *m*/*z* 727.2091 [M − H − gastrodin]^−^ which is derived from the deprotonated ion with molecular ion peak at *m*/*z* 995.3068 [M − H]^−^.

On the other hand, peaks **7** and **11** in Figure 2 showed deprotonated molecular ion peaks at *m*/*z* 429. 1396 [M − H]^−^, and *m*/*z* 697.23445 [M − H]^−^ and *m*/*z* 743.2399 [M − H + HOOH]^−^, respectively, and the molecular masses of peaks **7** and **11** were 30 amu less than those of parishin E and parishin B or C, respectively. The UV spectra (λmax 223.0 and 268.9 nm) of both compounds were very similar to those of parishins A, B and C (221.8 and 268.9 nm). These results indicate that the two compounds have chromophores that are similar to those of parishins A, B and C. Therefore, we attempted chromatographic isolation of the parishin-related compounds, including two novel compounds (**7**, **11**), for a definite structural identification based on spectroscopic data.

### 2.2. Isolation and Structure Elucidation of Parishin-Related Compounds

**Figure 3:**
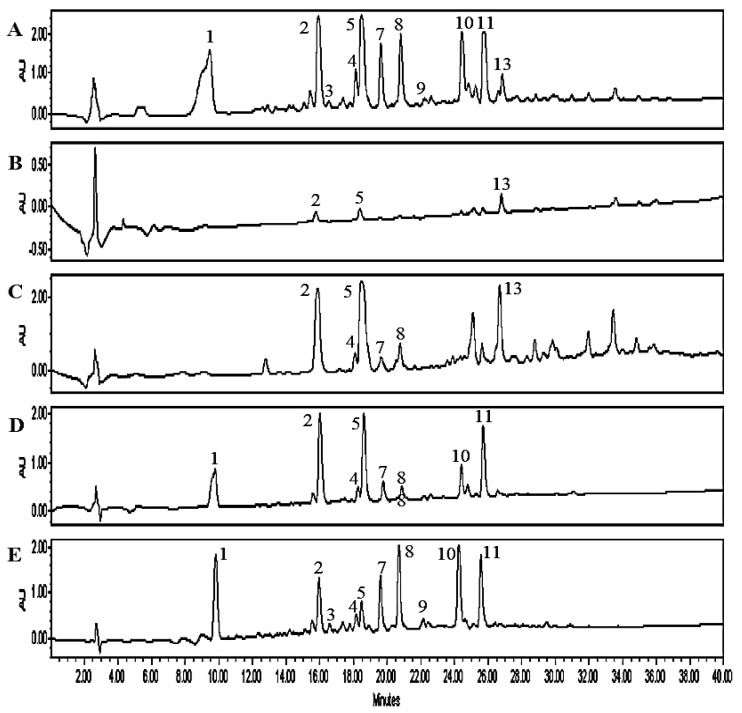
HPLC (220 nm) profile of solvent fractionation obtained from 70% MeOH extract of *M. tricuspidata* twig. (**A**) 70% MeOH extract; (**B**) *n*-hexane fraction; (**C**) ethyl acetate fraction; (**D**) *n*-butanol fraction; (**E**) water fraction; **1**, gastrodin; **2**, taxifolin-7-β-d-glucoside; **3**, parishin E; **4**, chlorogenic acid; **5**, dihydrokaempferol-7-β-d-glucoside; **7**, unknown-1; **8**, parishin B; **9**, parishin C; **10**, parishin A; **11**, unknown-2; **13**, kaempferol-3-β-d-glucoside.

**Scheme 1:**
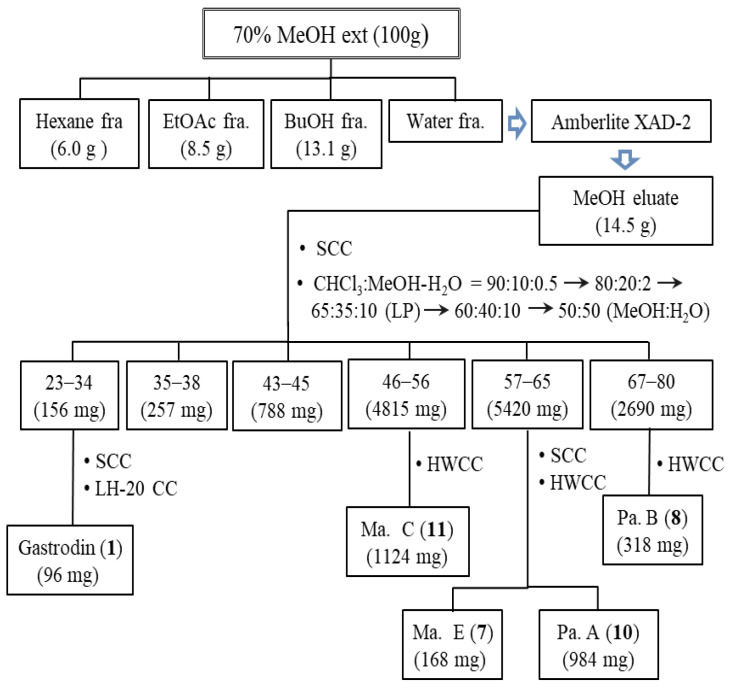
Isolation of parishin and its derivatives from *M. tricuspidata* twig. SCC, silica gel column chromatography (CC); HWCC, Toyopearl HW-40S CC; pa, parishin; Ma, macluraparishin.

The 70% MeOH extract of MT twig was suspended in water and was successively partitioned with *n*-hexane, ethyl acetate, water-saturated *n*-butanol and water fractions. Each solution was evaporated to obtain the concentrates from *n*-hexane, ethyl acetate, butanol and water fractions. The HPLC chromatograms of 70% MeOH extract and four concentrates are shown in Figure 3. Thirteen compounds were labelled in the range of retention time, 8–30 min of the chromatogram recorded at 220 nm. Among the detected compounds, the main compounds, taxifolin-7-β-d-glucoside (peak **2**) and dihydrokaempferol-7-β-d-glucoside (peak **5**) were mainly distributed in the ethyl acetate and butanol fractions. In contrast, parishin-related compounds including gastrodin (peak **1**), unknown-1 (peak **7**), parishin B (peak **8**), parishin A (peak **10**) and unknown-2 (peak **11**) were mainly detected in the water fraction as shown in Figure 3E. Polarity-driven partition methods have been widely applied in the extraction and separation of compounds from plant matrices. The distribution of these compounds in solvents of different polarity is related to the electronegativity of the chemical compounds [36]. Alcohols and acids are readily soluble in hydrophilic solvents as these compounds have high polar properties due the presence of an electronegative oxygen atom in their function groups [37]. The constituents in the water fractions were isolated by a combination of Amberlite XAD-2, silica gel, Toyopearl HW-40S and Sephadex LH-20 column chromatography, as shown in Scheme 1. 

Compound **1** (peak no. **1** in Figure 3E) was isolated as a white amorphous powder. The UV spectrum showed absorption maxima at 220.6 and 269.2 nm. ESI-MS showed deprotonated molecular ion peaks at *m*/*z* 331.1 [M − H + HCOOH]^−^, 571.1 [2M − H]^−^ (in negative mode), and 309.1 [M + Na]^+^, 595.2 [2M + Na]^+^ (in positive mode), respectively, corresponding to a molecular formula of C_13_H_18_O_7_ (molecular mass 286.28). ^1^H-NMR data (600 MHz, CD_3_OD) exhibited one anomeric proton signal at δ 4.88 (1H, d, *J* = 7.2 Hz, H-8) and signals of glucose moiety at δ 3.40−3.46 (m, 4H, H-9, 10, 11, 12) in addition to signals due to benzylic methylene at δ 3.88 (1H, dd, *J* = 12.0, 2.4 Hz, H-13a), 3.69 (1H, dd, *J* = 12.0, 5.4 Hz, H-13b) and aromatic proton signals at δ 7.27 (2H, d, *J* = 7.8 Hz, H-2, H-6) and δ 7.07 (2H, d, *J* = 9.6 Hz, H-3, H-5).

**Figure 4:**
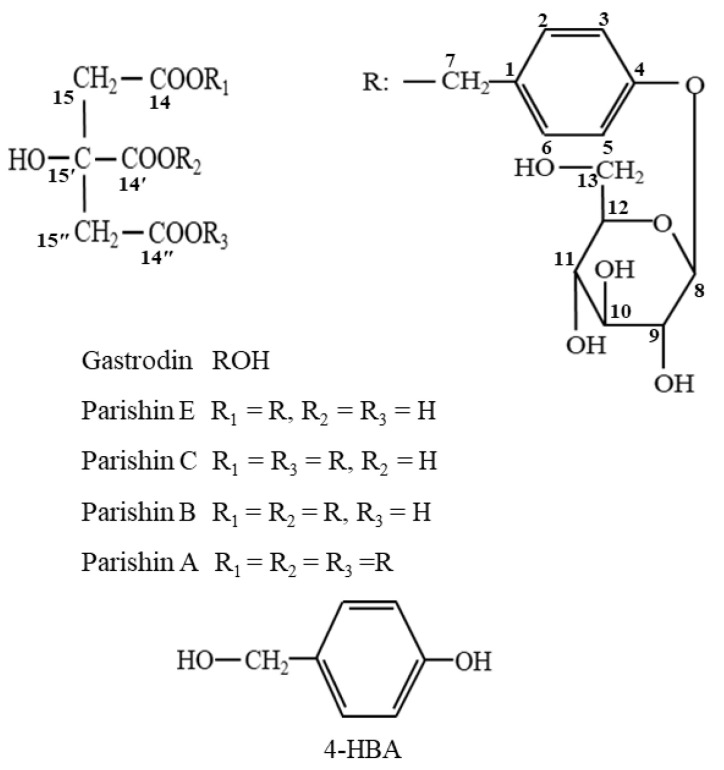
Chemical structures of parishin and its derivatives identified from MT twig.

In the ^13^C-NMR (150 MHz, CD_3_OD) spectrum, δ 129.6 (×2, C-2, 6) and 117.8 (×2, C-3, 5) indicated the presence of the *p*-disubstituted benzene ring. An oxy-methylene signal was observed at δ 65.0 (C-7) with 158.6 (C-4) and 136.7(C-1) indicating the presence of 4-HBA as well as signals due to the glucose moiety at 102.4 (C-8), 78.2 (C-10), 78.1 (C-12), 75.0 (C-9), 71.5 (C-11) and 62.6 (C-13). The glycosylation site of the glucose was observed at the phenolic hydroxyl group (C-4) from the chemical shifts of the C-1 and C-7 signals. From the above results, compound **1** was elucidated as 4-(β-d-glucopyranosyloxy)benzyl alcohol (gastrodin) as shown in Figure 4 [38,39,40].

Compound **8** (peak no. **8** in Figure 3E) was isolated as a white amorphous powder. The UV spectrum showed absorption maxima at 221.7 and 269.2 nm. The negative QTOF-MS spectrum showed a deprotonated molecular ion peak at *m*/*z* 727.2033 [M − H]^−^, corresponding to the molecular formula of C_32_H_40_O_19_ (molecular mass 728.65). The ^1^H-NMR (600 MHz, CD_3_OD) spectrum of compound **8** indicated the presence of two *p*-disubstituted benzene rings at δ 7.26 (2H, d, *J* = 8.1 Hz, H-2, 6), 7.21 (2H, d, *J* = 8.1 Hz, H-2′, 6′), 7.08 (2H, d, *J* = 8.3 Hz, H-3, 5) and 7.02 (2H, d, *J* = 8.3 Hz, H-3′, 5′), two hydroxyl methylene protons at δ 4.90 (2H, br. s, H-7a, 7b) and 4.87 (2H, br. s, H-7′a, 7′b) and two aromatic proton signals at δ 4.89 (2H, d, *J* = 7.5 Hz, H-8, 8′) indicating the presence of two gastrodin moieties in the molecule. The ^13^C-NMR spectrum (150 MHz, CD_3_OD) of compound **8** indicated the presence of three carbonyls at δ 175.1 (C-14′) and 171.2 (C-14, C-14′′), three aromatic carbon signals at δ 159.2 (×2, 4, 4′), 131.0 (×6, C-1, 1′, C-2, 2′, 6, 6′) and 117.8 (×4, 3, 3′, 5, 5′), two symmetrical glucose carbon signals at δ 102.3 (×3, C-8, 8′), 78.1 (×2, C-10, 10′), 78.0 (×2, C-12, 12′), 74.9 (×2, C-9, 9′), 74.8 (C-15′) and 71.4 (×2, H-11, 11), two benzylic carbon signals at δ 68.2 (C-7′), 67.3 (C-7) and 62.5 (×2, C-13, 13′) with two methylene carbon signals of the citric acid moiety at 44.9 (×2, C-15, 15′′). From the above results, compound **8** was identified as 1,2-bis [4-(β-d-glucopyranosyloxy)benzyl] citrate (parishin B) (Figure 4). Parishin B was identified previously in the *G. elata* rhizome [39,40,41,42]. These results suggest that the MT twig contained large amount of gastrodin, parishin A and parishin B. The parishin compounds and their metabolites gastrodin and 4-HBA have been recognized as important bioactive constituents in the traditional Chinese medicinal plant, *G. elata* rhizome, and have an anticonvulsant, analgesia, calmness, hypnosis, nootropic and anti-brain aging function for the central nervous system in the traditional therapy of Chinese medicine [2,25,26,27]. Therefore, MT twig might also have potential for pharmacological applications which are similar to those of *G. elata* rhizome.

Compound **10** was obtained as a white amorphous powder. The UV spectrum showed absorption maxima at 221.7 and 269.2 nm. The negative and positive ESI-MS spectra showed deprotonated molecular ion peaks at *m*/*z* 995.3 [M − H]^−^ and 1019.3 [M + Na]^+^, respectively, corresponding to a molecular formula of C_45_H_56_O_25._ (molecular mass 996.92). The ^1^H-NMR spectrum (600 MHz, CD_3_OD) of compound **10** indicated the presence of three *p*-disubstituted benzene rings at δ 7.26 (4H, d, *J* = 7.8 Hz, H-2, 2′′, 6, 6′′), 7.16 (2H, d, *J* = 8.4 Hz, H-2′, 6′), 7.07 (4H, d, *J* = 8.7 Hz, H-3, 3′′, 5, 5′′), 7.04 (2H, d, *J* = 7.8 Hz, H-3′, 5′), three hydroxyl methylene protons at δ 4.98 (2H, s, H-7′), 4.88 (4H, s, H-7, 7′′), and three aromatic proton signals at δ 4.91 (3H, d, *J* = 7.2 Hz, H-8, 8′, 8′′) to indicate the presence of three gastrodin moieties in the molecule. In addition, two allyl methylene protons were observed at δ 2.94 (2H, d, *J* = 15.6 Hz, H-15a, 15″a) and 2.77 (2H, d, *J* = 15.0 Hz, H-15b, 15″b). The ^13^C-NMR spectrum (150 MHz, CD_3_OD) of compound **3** indicated the presence of three carbonyls at δ 174.5 (C-14′), 171.1 (×2, C-14, 14′′), three symmetrical glucose carbon signals at δ 102.3 (×3, C-8, 8′, 8′′), 78.2 (×3, C-10, 10′, 10′′), 77.9 (×3, C-12, 12′,12′′), 75.0 (×3, C-9, 9′, 9′′), 71.4 (×3, H-11, 11′, 11′′), 68.4 (C-7′) and 62.5 (×3, C-13, 13′, 13′′), three aromatic carbon signals at δ 159.2 (×3, C-4, 4′, 4′′), 131.2 (×6, C-2, 2′, 2′′, 6, 6′, 6′′), 131.2 (×2, C-1, 1′), 130.8 (C-1′) and 117.9 (×6, C-3, 3′, 3′′, 5, 5′, 5′′), two methylene carbon signals of the citrate moiety at δ 44.9 (×2, C-15, 15′′), two benzylic carbon signals at δ 67.5 (×2, C-7, 7′′). By comparing the above results and the previously reported spectroscopic data [38,39,40,42], compound **10** was identified as tris [4-(β-d-glucopyranosyloxy)benzyl] citrate, namely, parishin or parishin A (Figure 4). Parishin A has only been found in *G. elata* rhizome [38,39,40,42] and *Vanda parishii* [43].

### 2.3. Structural Elucidation of Novel Parishin Compounds

**Figure 5:**
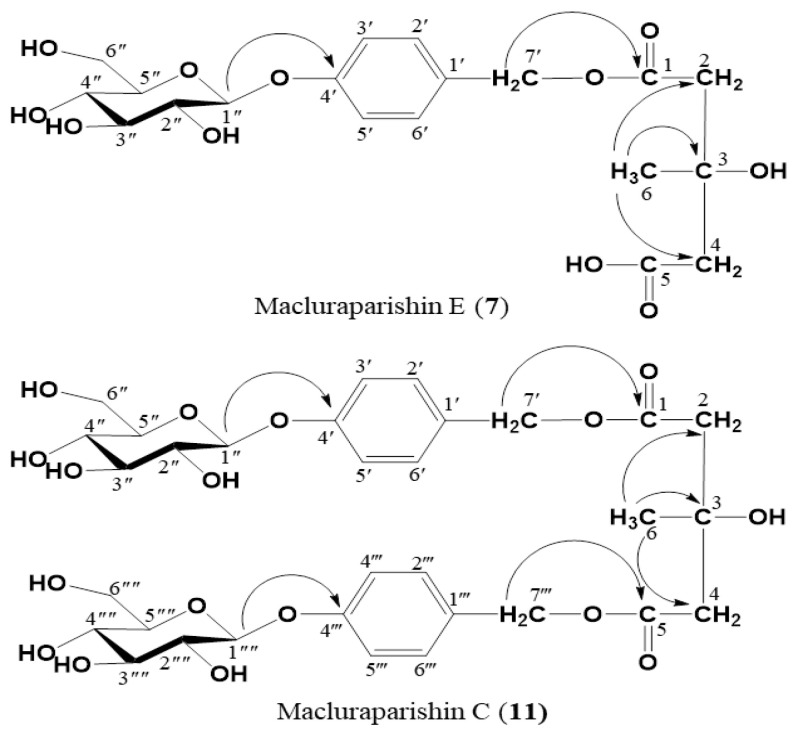
Key correlations in the HMBC experiments and chemical structures of novel parishin compounds identified from MT twig.

Compound **7** (peak no. **7** in Figure 3E) was isolated as a white amorphous powder from the water fraction by repeated silica gel and Toyopearl HW-40S column chromatography and the molecular formula was determined to be C_19_H_26_O_11_ based on the deprotonated molecular ion peak [M − H] observed at *m*/*z* 429.1391 (calcd for C_19_H_25_O_11_, 429.1397, error value ∆ = −1.4 ppm) in negative QTOF-MS. The ^13^C-NMR (150 MHz, CD_3_OD) and ^1^H-NMR (600 MHz, CD_3_OD) spectra of compound **7** are very similar to those of 1-(4-β-d-glucopyranosyloxybenzyl)citrate (named parishin E) [44,45], except one carboxyl group in the citric acid moiety was substituted by a methyl group (δ_C_ 27.7; δ_H_ 1.31, 3H, s, C-6). The NMR spectra of this compound showed one oxygenated-olefin quaternary (δ_C_ 131.4, C-1′), four olefin methine [(δ_C_ 117.7; δ_H_ 7.08, 2H, d, *J* = 8.4 Hz, C-3′, 5′) and (δ_C_ 130.9; δ_H_ 7.30, 2H, d, *J* = 8.4 Hz, C-2′, 6′)], one olefin quaternary (δ_C_ 159.0, C-4′) and one oxygenated-methylene (δ_C_ 66.9; δ_H_ 5.05, 2H, s, C-7′) signals, due to the presence of 4-HBA as an aglycone moiety. In addition, signals were also observed for one hemiacetal (δ_C_ 102.2; δ_H_ 4.91, 1H, d, *J* = 7.8 Hz, C-1″), four oxygenated-methine [(δ_C_ 74.9; δ_H_ 3.47, 1H, dd, *J* = 7.8, 6.6 H, C-2′′), (δ_C_ 77.9; δ_H_ 3.54, 1H, dd, *J* = 6.6, 6.6 Hz, C-3′′), (δ_C_ 71.3; δ_H_ 3.42, 1H, dd, *J* = 8.4, 6.6 Hz, C-4′′) and (δ_C_ 78.1; δ_H_ 3.44, 1H, ddd, *J* = 8.4, 4.8, 2.4 Hz, C-5′′)] and one oxygenated-methylene (δ_C_ 62.5; δ_H_ 3.88, 1H, dd, *J* = 12.6, 2.4 Hz; δ_H_ 3.70, 1H, dd, *J* = 12.6, 4.8 Hz, C-6′′), and these signals were due to the presence of a glucosyl moiety in the molecule. The NMR spectra of compound **7** also showed signals due to one carboxyl group (δ_C_ 177.5, C-5), one ester carbon (δ_C_ 172.7, C-1), one oxygenated-methine (δ_C_ 70.9, C-3), two methylene [(δ_C_ 46.9; δ_H_ 2.64, 2H, s, C-2), (δ_C_ 46.7; δ_H_ 2.56, 1H, d, *J* = 15.0 Hz; δ_H_ 2.48, 1H, d, *J* = 15.0 Hz, C-4)] and one methyl group (δ_C_ 27.7; δ_H_ 1.31, 3H, s, C-6). These results led us to postulate the presence of 4-HBA, glucose and an organic acid-bearing methyl group in its molecule (Table 2). The evidence that the primary alcohol (C-7′) of 4-HBA in the gastrodin moiety is bound to the C-1 carboxyl group of the acid moiety is due to the correlation between H-7′ and C-1 in the HMBC spectrum (Figure 5). Furthermore, the chemical shift values in the ^13^C-NMR spectrum of this compound indicated that a 3-hydroxyl-3-methyl-pentane-1,4-dioic acid moiety was present, which was represented by characteristic ^13^C-NMR signals at δ_C_ 27.7 (C-6), 46.7 (C-4), 46.9 (C-2), 70.9 (C-3), 172.7 (C-1) and 177.5 (C-5) [46,47]. The evidence that the methyl group is acylated to the C-3 position in the acid moiety is also due to the correlation between H-6/C-2, H-6/C-3 and H-6/C-4 in the HMBC spectrum. The evidence that the glucopyranosyl group is bound to the phenolic hydroxyl group of 4-HBA is due to the correlation between the anomeric proton signal (H-1′′) of the glucosyl moiety with the phenolic hydroxyl group (C-4′) of 4-HBA in the HMBC spectrum. Based on these results, compound **7** was determined to be 1-[4-β-d-glucopyranosyloxybenzyl]-3-hydroxy-3-methyl-pentane-1,5-dioate (named macluraparishin E), as shown in Figure 5. The substance 3-Hydroxy-3-methyl-pentane-1,5-dioic acid which is also known as 3-hydroxy-3-methylglutaric acid or meglutol has been rarely found in the acylated forms with flavonoid glycosides in plant families such as Leguminosae, Liliaceae, Polygalaceae and Rosacea [46,47].

Compound **11** was isolated as a white amorphous powder and the molecular formula was determined to be C_32_H_42_O_17_ based on the deprotonated molecular ion peak [M − H]^−^ at *m*/*z* 697.2352 (calcd for C_32_H_41_O_17_, 697.2344, ∆ = +1.3 ppm) in negative QTOF-MS. The fragment ion peak (*m*/*z* 429.1401) observed in QTOF-MS could be regarded as a fragment ion due to the loss of one gastrodin moiety from the molecule. The ^13^C- (150 MHz, CD_3_OD) and ^1^H-NMR (600 MHz, CD_3_OD) spectra of this compound were similar to those of compound **7**, except that an additional gastrodin moiety was bound with the carbonyl group (C-5) in the acid moiety and they were also similar to those of 1,3-bis [4-(β-d-glucopyranosyloxy)benzyl]citrate (named parishin C) except that one carboxyl group in the citric acid moiety was substituted by a methyl group (δ_C_ 28.0; δ_H_ 1.32, 3H, s, C-6). In the chemical structure of compound **11**, it was also observed that two molecules of gastrodin were bound in a symmetrical structure in the two carboxyl groups (C-1 and C-5) of the acid moiety. In addition, two oxygenated-olefin quaternary (δ_C_ 159.1 × 2, C-4′, 4′′′), eight olefin methine (δ_C_ 131.0 × 2; δ_H_ 7.28, 4H, d, *J* = 8.4 Hz, C-2′, 6′, 2′′′, 6′′′)] and [(δ_C_ 117.7 × 2; δ_H_ 7.07, 4H, d, *J* = 8.4 Hz, C-3′, 5′, 3′′′, 5′′′), two olefin quaternary (δ_C_ 131.4 × 2, C-1′, 1′′′) and two oxygenated-methylene (δ_C_ 67.0 × 2; δ_H_ 5.03, 4H, s, C-7′, 7′′′) signals were observed in the NMR spectral data (Table 2).

These signals are due to the presence of 4-HBA as the aglycone part of gastrodin. Furthermore, 2 hemiacetal (δ_C_ 102.2 × 2; δ_H_ 4.90, 2H, d, *J* = 7.8 Hz, C-1′′, 1′′′′), 8 oxygenated-methine [(δ_C_ 74.9 × 2; δ_H_ 3.44, 2H, dd, *J* = 7.8, 6.6 Hz, C-2′′, 2′′′′), (δ_C_ 78.0 × 2; δ_H_ 3.46, 2H, dd, *J* = 6.6, 6.6 Hz, C-3′′, 3′′′′), (δ_C_ 71.4 × 2; δ_H_ 3.40, 2H, dd, *J* = 8.4, 6.6 Hz, C-4′′, 4′′′′) and (δ_C_ 78.2 × 2; δ_H_ 3.43, 2H, ddd, *J* = 8.4, 5.4, 2.4 Hz, C -5′′, 5′′′′)], 2 oxygenated-methylene (δ_C_ 62.5 × 2; δ_H_ 3.88, 2H, dd, *J* = 12.6, 2.4 Hz; δ_H_ 3.69, 2H, dd, *J* = 12.6, 5.4 Hz, C-6′′, 6′′′′) signals were observed. These results indicate that compound **11** contained two β-d-glucopyranosyl units. Significant correlation was also observed between H-6/C-2, H-6/C-3 and H-6/C-4 in the HMBC spectrum (Figure 5). These results suggest that the carboxyl group at the C-3 position of the acid moiety is substituted by a methyl group. The observation of the signals of two ester carbon at δ_C_ 172.4 (×2, C-1, 5), one oxygenated-methine at δ_C_ 70.9 (C-3), two methylene (×2, δ_C_ 46.3; δ_H_ 2.67, 4H, d, *J* = 6.0 Hz, C-2, 4) and one methyl (δ_C_ 28.0; δ_H_ 1.32, 3H, s, C-6) are due to the presence of the acid moiety. The correlation was observed between the two primary alcohols (H-7′, 7′′′) of 4-HBA and the two carboxyl groups (C-1, 5) in the HMBC spectrum. These results indicate that the two primary alcohols (C-7′, C-7′′′) of 4-HBA were combined with two carboxyl groups (C-1, C-5) of the acid moiety. The evidence that the two glucopyranosyl groups are bound to the phenolic hydroxyl groups of two 4-HBA are due to the correlation between the anomeric proton signals (H-1′′, 1′′′) of the glucosyl moieties with the phenolic hydroxyl groups (C-4′, C-4′′′) of two 4-HBA in the HMBC spectrum. From these results, compound **11** was determined to be 1,3-bis [4-β-d-glucopyranosyloxy)benzyl]- 3-hydroxy-3-methyl-pentane-1,5-dioate (named macluraparishin C). To the best of our knowledge, macluraparishins E and C are identified as plant-based phytochemicals in this study for the first time. Parishin A is an ester compound formed by the condensation of gastrodin moieties of three moles with citric acid, and parishins B, C and E have one or two less moieties of gastrodin than parishin A. Parishin A is metabolized into gastrodin via parishins B, C or E, to finally be processed into 4-HBA, glucose and citric acid in vitro and in vivo systems [48,49,50]. Two novel parishins (macluraparishins E and C) are ester compounds formed by the condensation of gastrodin moieties of one or two moles with 3-hydroxy-3-methyl-pentane-1,5-dioic acid. Therefore, macluraparishins E and C may also be metabolized into 4-HBA, glucose and 3-hydroxy-3-methyl-pentane-1,5-dioic acid. The substance 3-Hydroxy-3-methyl-pentane-1,5-dioic acid is known to be a competitive inhibitor of 3-hydroxy-3-methylglutaryl-CoA reductase and strongly reduces cholesterol, triglycerides, serum β-lipoproteins and phospholipids in vitro and in vivo systems [51,52].

### 2.4. Comparison of Parishin Compounds in Different Parts of MT

The compositions and contents of plant-based phytochemicals vary significantly between different parts of the plants (i.e., bark, root, leaves, stem, fruit and seed), thereby having their own characteristic bioactivities in in vitro and in vivo systems. The MT also has different bioactivities and chemical compositions depending on the parts of the plant [6]. Six parishins and the metabolite (gastrodin) from the different parts (i.e., twig, bark, root, leaves, xylem and fruit) of MT were quantified for the first time based on the peak areas of the chromatograms recorded at 220 nm. The contents of individual constituents are presented in Table 3.

Gastrodin was detected as a dominant constituent in all parts of MT except in the fruit. The twig had the highest amount of gastrodin (7420.0 ± 150.8 µg/g, dw), followed by the root (2470.44 ± 10.6 µg/g), bark (2442.2 ± 25.5 µg/g), leaves (1758.4 ± 14.3 µg/g) and xylem (1397.1 ± 104.2 µg/g). Furthermore, parishin A was abundant in the twig (2498.1 ± 90.0 µg/g), bark (3234.8 ± 78.6 µg/g) and fruit (1954.3 ± 10.1 µg/g)), while parishin B was abundant in the twig (3744.8 ± 63.5 µg/g), bark (3555.0 ± 87.0 µg/g) and root (1291.8 ± 25.7 µg/g). Macluraparishins C (2639.1 ± 63.1 − 3151.1 ± 17.5 µg/g) and macluraparishin E (2567.4 ± 75.6 − 4341.4 ± 24.4 µg/g) were abundant in the twig, bark and root. In contrast, these constituents in the xylem and fruit parts were present as small amounts or not detected at all and parishin A and its derivatives, apart from gastrodin, were not detected in the leaves. MT twig, bark and root might have high potential as a substitute for the traditional herbal medicine, *G. elata* rhizome, as they contained high amounts of parishin-related compounds.

## 3. Materials and Methods

### 3.1. Plant Materials

Amounts of 3 kg each of hot-air dried (65 °C) samples (twig, leaves, root and stem) and fresh frozen fruit as different parts of MT plant were directly purchased from a local farm in Milyang city, gyeongsangnam-do, South Korea, in late March 2021. The samples were authenticated by Professor Byung-Kil Choo (Department of Crop Agriculture and Life Science, Jeonbuk National University, Jeonbuk, Republic of Korea). Voucher specimens (FL-202101−FL-202105) were stored in the Laboratory of Fermentation Technology (Professor Myung-Kon Kim, Jeonbuk National University, Jeonbuk, Republic of Korea). The stem was peeled manually and divided into bark and xylem parts. The frozen fruit was washed with tap water, followed by freeze-drying. The samples were pulverized using a household grinder (Hanil SFM-700SS, Yeongdeungpo-gu, Seoul, Republic of Korea). The pulverized samples were kept in air-tight plastic containers and stored in a cold room (4 °C) until use.

### 3.2. Reagents and Chemicals

Sephadex LH-20 resin was purchased form Sigma-Aldrich (St. Louis, MO, USA). Gastrodin, parishins A, B, C and E were purchased from Chengdu Biopurify Phytochemicals Ltd. (Chengdu, Sichuan, China). Toyopearl HW-40S polymer was purchased from Tosoh Corp. (Tokyo, Japan). Silica gel F_254_ TLC plate, silica gel (230–400 mesh) for column chromatography, CD_3_OD and tetramethylsilane (TMS) were purchased from Merck KGaA (Darmstadt, Germany). Deionized water and methanol for HPLC were purchased from J.T. Baker (Center Valley, PA, USA). Other reagents used were of analytical grade and purchased from commercial sources (Daihan Scientific Co., Ltd., Wonju, Gangwon-do, Republic of Korea).

### 3.3. Isolation of Parishin Compounds in MT Twig

The powdered MT twig (1.0 kg) was extracted with 3 L of 70% aqueous MeOH with an ultrasonicator (Hwashin Instrument Co., Seoul, Korea) at room temperature for 20 min, followed by centrifugation at 4500 rpm for 15 min. The residue was further extracted twice with 2 L each of 70% aqueous methanol followed by centrifugation as above. The supernatants were combined and evaporated under reduced pressure to obtain MeOH extract (105 g; yield 10.5%). The MeOH extract (100 g) was suspended in water (500 mL) and sequentially extracted with *n*-hexane, ethyl acetate and water-saturated *n*-butanol (each 500 mL × 3). Each fraction was evaporated under reduced pressure to obtain *n*-hexane (6.0 g), ethyl acetate (8.5 g), *n*-butanol (13.0 g) and water fractions, respectively. The water fraction was passed through preconditioned Amberlite XAD-2 column (50 × 5 cm i.d.) at a rate of 10 mL/min and was washed with distilled water (1.5 L) to remove sugars and organic acids. Parishin and its derivatives were eluted from the column with MeOH (1.5 L). The eluate was evaporated under reduced pressure to obtain the water fraction containing mainly parishin and its derivatives (14.5 g). The water fraction (14 g) was separated by column chromatography on silica gel (300 g) and stepwise elution with a mixture of CHCl_3_−MeOH−H_2_O (90:10:0.5, 80:20:1, 65:35:10, lower phase, 50:50:0) to yield 6 fractions (MF-1−MF-6). Fra. MF-1 eluted with CHCl_3_−MeOH−H_2_O (80:20:2) was further separated by silica gel chromatography and eluted with a stepwise gradient of CHCl_3_−MeOH (100:0 to 70:30) to obtain gastrodin (96 mg). Fra. MF-4 eluted with CHCl_3_−MeOH−H_2_O (60:40:10) was purified by column chromatography using Toyopearl HW 40S polymer to obtain macluraparishin C (1124 mg). Fra. MF-5 eluted with MeOH−H_2_O (50:50) was firstly separated by silica gel column chromatography with CHCl_3_−MeOH−H_2_O (65:35:10, lower phase) and then purified by Toyopearl HW 40S column chromatography with 70% MeOH to obtain parishin A (984 mg) and macluraparishin E (168 mg). Fra. MF-6 eluted with MeOH−H_2_O (50:50) was further purified by Toyopearl HW 40S column chromatography with 70% MeOH to obtain parishin B (318 mg.). The overall isolation procedure of compounds is presented in Scheme 1.

Gastrodin (1): colorless powder; UV (MeOH) λ_max_ (nm) 220.6, 269.2; negative-ESI-MS *m*/*z* 331 [M − H + HCOOH]¯, 571[2M − H]¯; positive ESI-MS m/z309 [M + Na]^+^ *m*/*z* 595 [2M + Na]^+^; ^1^H-NMR (600 MHz, CD_3_OD) δ 7.27 (2H, d, *J* = 7.8 Hz, H-2, 6), 7.07 (2H, d, *J* = 9.6 Hz, H-3, 5), 4.88 (1H, d, *J* = 7.20 Hz, H-8), 3.88 (1H, dd, *J* = 12.0, 2.4 Hz, H-13a), 3.69 (1H, dd, *J* = 12.0, 5.4, 12.0 Hz, H-13b), 3.40–3.46 (m, 4H, H-9, 10, 11, 12); ^13^C-NMR (600 MHz, CD_3_OD) δ 158.6 (C-4), 136.7 (C-1), 129.6 (×2, C-2, 6), 117.8 (×2, C-3, 5), 102.5 (C-8), 78.2 (C-10), 78.1 (C-12), 75.0 (C-9), 71.5 (C-11), 65.0 (C-7), 62.6 (C-13).

Parishin B (**8**): colorless powder; UV (MeOH) λ_max_ (nm) 221.8, 268.9; negative ESI-MS *m*/*z* 727.6 [M − H]^−^; ^1^H-NMR (600 MHz, CD_3_OD) δ 7.26 (2H, d, *J* = 8.1 Hz. H-2, 6), 7.21 (2H, d, *J* = 8.1 Hz, H-2′, 6′), 7.08 (2H, d, *J* = 8.3 Hz, H-3, 5), 7.02 (2H, d, *J* = 8.3 Hz, H-3′, 5′), 4.90 (2H, br. s, H-7a, 7b), 4.89 (2H, d, *J* = 7.5 Hz, H-8, 8′), 4.87 (2H, br. s, H-7′a, 7′b), 3.88 (2H, dd, *J* = 12.0, 1.8 Hz, H13a, 13′a), 3.70 (2H, dd, *J* = 11.9, 4.7 Hz, H-13b, 13′b), 2.93 (1H, d, *J* = 15.6 Hz, H-15a), 2.88 (1H, d, *J* = 15.6 Hz, H-15′′a), 2.82 (1H, d, *J* = 15.6 Hz, H-15b), 2.67 (2H, d, *J* = 15.6 Hz, H-15′′b); ^13^C-NMR (150 MHz, CD_3_OD) δ 175.1 (C-14′), 171.2 (×2, C-14, C-14′′), 159.2 (×2, 4, 4′), 131.0 (×2, C-2, 2′), 131.0 (×2, C-1, 1′), 130.1 (×2, 6, 6′), 117.8 (×4, 3, 3′, 5, 5′), 102.3 (×2, 8, 8′), 78.1 (×2, 10, 10′), 78.0 (×2, 12, 12′), 74.9 (×2, 9, 9′), 74.8 (C-15′), 71.4 (×2, C-11, 11′), 68.2 (C-7′), 67.3 (C-7), 62.5 (×2, C-13, 13′), 44.9 (×2, C-15, 15′′).

Parishin A (**10**): colorless powder; UV (MeOH) λ_max_ (nm) 221.7, 269.2; negative ESI-MS *m*/*z* 995.1 [M − H]^−^, positive ESI-MS *m*/*z* 1019.3 [M + Na]^+^; ^1^H-NMR (600 MHz, CD_3_OD) δ 7.26 (4H, d, *J* = 7.8, Hz, H-2,2′′, 6, 6′′), 7.16 (2H, d, 2H, *J* = 8.4 Hz, H-2′, 6′), 7.07 (4H, d, *J* = 8.7 Hz, H-3, 3′′, 5, 5′′), 7.04 (2H, d, *J* = 7.8 Hz, H-3′, 5′), 4.98 (2H, H-7′), 4.91 (d, 3H, *J* = 7.2 Hz, H-8, 8′, 8′′), 4.88(4H, s, H-7, 7′′), 3.87 (3H, dd, *J* = 12.3, 1.8 Hz, H-13a,13′a,13′′a), 3.70 (3H, dd, *J* = 12.9, 5.4 Hz, H-13b,13′b,13′′b), 3.40–3.48 (12H, m, D-glucopyranosyl moiety), 2.94 (2H, d, *J* = 15.6 Hz, H-15a, 15′′a), 2.77 (2H, d, *J* = 15.0 Hz, H-15b, 15′′b); ^13^C-NMR (150 MHz, CD_3_OD) δ 174.5 (C-14′), 171.1 (×2, C-14, 14′′), 159.2 (×3, C-4, 4′, 4′′), 131.2 (×6, C-2, 2′, 2′′, 6, 6′, 6′′), 131.2 (×2, C-1, 1′′), 130.8 (C-1′), 117.9 (×6, C-3, 3′, 3′′, 5, 5′, 5′′), 102.3 (×3, C-8, 8′, 8′′), 78.2 (×3, C-10, 10′, 10′′), 77.9 (×3, C-12, 12′, 12′′), 75.0 (×3, C-9, 9′, 9′′), 74.8 (C-15′), 71.4 (×3, C-11, 11′, 11′′), 68.4 (C-7′), 67.5 (×2, C7, 7′′), 62.5 (×3, C-13, 13′, 13′′), 44.9 (×2, C-15, 15′′).

### 3.4. Sample Extraction, HPLC and HPLC-QTOF-MS Analysis

Powdered samples (1.0 g) were extracted thrice with 20 mL of 70% aqueous MeOH using ultrasonicator (Hwashin Instrument Co., Seoul, Republic of Korea) at room temperature for 20 min. The extract was centrifuged at 9000× *g* for 20 min. The supernatant was evaporated under reduced pressure, dissolved in 10 mL of 70% aqueous MeOH and filtered using membrane filter (0.45 um). HPLC analysis was performed using an HPLC system (Waters, Milford, MA, USA) equipped with a 600E system controller, 717 plus autosampler and Waters 996 PDA with a Zorbax Eclipse XDB C_18_ column (4.6 mm × 250 mm, 5 μm; Agilent Technologies, Inc., Santa Clara, CA, USA). The mobile phase consisted of 0.1% formic acid in water (A) and MeOH (B). The ratio of mobile phase was maintained at A:B = 95:5 (*v*/*v*) (0–5 min), 85:15 (5–10 min), 45:55 (10–25 min) and 95:10 (25–40 min) at a flow rate of 0.8 mL/min. The amount of each constituent was quantified using a concentration plot (12.5−400 ug/mL) of authentic standards based on peak area. All experiments were performed in triplicate, and the data were expressed as the mean ± standard deviation. HPLC-QTOF-MS analysis was carried out according to the previously reported method [24].

### 3.5. General Experimental Conditions

HPLC−MS (negative and positive mode) was conducted on an Agilent 1100 mass spectrometer (Santa Clara, CA, USA) with an electrospray ionization (ESI) in negative ion mode. The high resolution QTOF-MS was performed with Waters Synapt G2-Si HMDS instrument (Waters Corp., Milford, MA, USA) with an ESI in negative and positive ion modes. NMR spectra were taken on a JEOL model JNM−ECA 600 FT−NMR spectrometer (Akishima, Tokyo, Japan) at 600 MHz (^1^H-NMR) and 150 MHz (^13^C-NMR) in CD_3_OD with tetramethylsilane as an internal standard.

## 4. Conclusions

In this study, a phytochemical study of the MT plant revealed the presence of several parishin-related compounds including gastrodin, parishins A, B, C, and two novel parishin derivatives (named macluraparishins E and C) possessing 3-hydroxy-3-methyl-pentane-1,5-dioic acid as an acid moiety, based on the spectroscopic evidence. The parishin-related compounds and their metabolites (gastrodin and 4-HBA) have been recognized as important bioactive constituents in the herbal medicine obtained from *G. elata* rhizome, which has been used for the prevention and treatment of the brain and central nervous system disorders as an anticonvulsant and for analgesia, calmness, hypnosis and brain aging in traditional Oriental therapy. In particular, the twig, bark and root of MT contained a large amount of macluraparishins E and C, identified for the first time in the study as plant-based phytochemicals. Therefore, these results indicate that the twig, bark and root of the MT plant could be used as a potential pharmacological agent similar to that of the *G. elata* rhizome.

## Figures and Tables

**Table 1:** Tentative identification of polyphenols in 70% MeOH extract of MT twig by HPLC-QTOF-MS.

Peak no.	RT (min)	[M − H]^−^ or [M + HCOO]^−^ (*m*/*z*)	Molecular Formula	Error (ppm)	MW (*m*/*z*)	Identification
1	9.20	331.1031	C_13_H_18_O_7_	1.02	332.11	Gastrodin
2	17.77	465.1031	C_21_H_22_O_12_	0.73	466.11	Taxifolin-7-β-d-glucoside
3	18.46	459.1138	C_19_H_24_O_13_	0.68	460.12	Parishin E
4	19.35	353.0872	C_16_H_18_O_9_	1.55	354.09	Chlorogenic acid
5	19.96	449.1082	C_21_H_22_O_11_	0.14	450.12	Dihydrokaempferol-7-β-d- glucoside
6	20.49	625.1405	C_27_H_30_O_17_	0.89	626.15	Quercetin-3-β-d-sophoroside
7	21.16	429.1396	C_19_H_26_O_11_	0.81	430.15	Unknown-1
8	21.54	727.2085	C_32_H_40_O_19_	0.72	728.22	Parishin B
9	22.46	727.2086	C_32_H_40_O_19_	0.59	728.22	Parishin C
10	23.71	995.3025	C_45_H_56_O_25_	1.16	996.31	Parishin A
11	24.83	743.2399	C_33_H_44_O_19_	0.61	744.25	Unknown-2
12	27.06	287.0558	C_15_H_12_O_6_	0.71	288.06	Dihydrokaempferol
13	27.53	447.0927	C_21_H_20_O_11_	1.37	448.10	Kaempferol-3-β-d-glucoside

**Table 2:** The ^13^C- and ^1^H-NMR data for macluraparishins E and C.

No.	Macluraparishin E (7)		Macluraparishin C (11)	
	δ_C_	δ_H_ (*J*_Hz_)	δ_C_	δ_H_ (*J*_Hz_)
1	172.7	-	172.4	
2	46.9	2.64 (2H, s)	46.3	2.67 (2H, d, 6.0)
3	70.9	-	70.9	-
4	46.7	2.56 (1H, d, 15.0) 2.48 (1H, d, 15.0)	46.3	2.67 (2H, d, 6.0)
5	177.5	-	172.4	-
6	27.7	1.31 (3H, s)	28.0	1.32 (3H, s)
1′	131.4	-	131.4	-
2′/6′	130.9	7.30 (2H, d, 8.4)	131.0	7.28 (2H, d, 8.4)
3′/5′	117.7	7.08 (2H, d, 8.4)	117.7	7.07 (2H, d, 8.4)
4′	159.0	-	159.1	-
7′	66.9	5.05 (2H, s)	67.0	5.03 (2H, s)
1′′	102.2	4.91 (1H, d, 7.8)	102.2	4.90 (2H, d, 7.8)
2′′	74.9	3.47 (1H, dd, 7.8, 6.6)	74.9	3.44 (2H, dd, 7.8, 6.6)
3′′	77.9	3.54 (1H, dd, 6.6, 6.6)	78.0	3.46 (2H, dd, 6.6, 6.6)
4′′	71.3	3.42 (1H, dd, 8.4, 6.6)	71.4	3.40 (2H, dd, 8.4, 6.6)
5′′	78.1	3.44 (1H, ddd, 8.4, 4.8, 2.4)	78.2	3.43 (2H, ddd, 8.4, 5.4, 2.4)
6′′	62.5	3.88 (1H, dd, 12.6, 2.4) 3.70 (1H, dd, 12.6, 4.8)	62.5	3.88 (2H, dd, 12.6, 2.4) 3.69 (2H, dd, 12.6, 5.4)
1′′′			131.4	-
2′′′/6′′′			131.0	7.28 (4H, d, 8.4)
3′′′/5′′′			117.7	7.07 (4H, d, 8.4)
4′′′			159.1	-
7′′′			67.0	5.03 (2H, s)
1′′′′			102.2	4.90 (2H, d, 7.8)
2′′′′			74.9	3.44 (2H, dd, 7.8, 6.6)
3′′′′			78.0	3.46 (2H, dd, 6.6, 6.6)
4′′′′			71.4	3.40 (2H, dd, 8.4, 6.6)
5′′′′			78.2	3.43 (2H, ddd, 8.4, 5.4, 2.4)
6′′′′			62.5	3.88 (2H, dd, 12.6, 2.4) 3.69 (2H, dd, 12.6, 5.4)

**Table 3:** Comparative analysis of parishin-related compounds from different parts of MT.

Peak No.	Compound	Twig	Bark	Root	Leaves	Xylem	Fruit
1	Gastrodin	7420.0 ± 150.8	2442.2 ± 25.5	2470.4 ± 10.6	1758.4 ± 14.3	1397.1 ± 104.2	855.9 ± 5.4
3	Parishin E	330.9 ± 9.9	466.4 ± 28.6	283.0 ± 11.2	-	42.3 ± 10.5	26.1 ± 7.2
7	Macluraparishin E	2567.4 ± 75.5	4341.4 ± 24.4	3558.1 ± 21.5	-	289.1 ± 66.3	121.7 ± 9.6
8	Parishin B	3744.8 ± 63.5	3555.0 ± 87.0	1291.8 ± 25.7	-	331.9 ± 23.5	-
9	Parishin C	-	381.3 ± 8.6	148.3 ± 40.4	-	14.5 ± 1.6	76.8 ± 1.5
10	Parishin A	2498.1 ± 90.0	3234.8 ± 78.6	1490.2 ± 46.1	-	461.8 ± 7.9	1954.3 ± 10.1
11	Macluraparishin C	2639.1 ± 63.1	3104.3 ± 29.2	3151.1 ± 17.5	-	202.0 ± 53.4	84.2 ± 0.4

Peak numbers refer to Figure 3A. The values (µg/g, DW) are mean ± standard deviation (*n* = 3).

## Data Availability

The data that support the findings of this study are available from the corresponding author (M.-K.K).

## Sample Availability

Samples of the compounds are available from the corresponding author (M.-K.K.).
